# Consumer and community involvement: implementation research for impact (CCIRI) – implementing evidence-based patient and public involvement across health and medical research in Australia – a mixed methods protocol

**DOI:** 10.1186/s12961-025-01293-0

**Published:** 2025-02-24

**Authors:** Ashley H. Ng, Sandra Reeder, Angela Jones, Ainslie Cahill, Debra Langridge, Susanne Baker, Leah Heiss, Alan Dorin, Helena Teede

**Affiliations:** 1 Monash Centre for Health Research and Implementation, Monash University and Monash Health, Melbourne, VIC Australia; 2 Monash Partners Academic Health Science Centre, Melbourne, VIC Australia; 3 Western Australia Health Translation Network, the University of Western Australia, Perth, Australia; 4 Monash Art Design and Architecture, Monash University, Melbourne, VIC Australia; 5 Faculty of Information Technology, Monash University, Melbourne, VIC Australia

**Keywords:** Public and patient involvement, Consumer and community involvement, Protocol, Implementation science, Co-design, Network

## Abstract

**Background:**

Within Australia, there is increasing recognition of the importance and value of patient and public involvement, or consumer and community involvement (CCI), in health and medical research and healthcare improvement. Despite this and policy mandates, there has been little behavioural and systems change to embed and support CCI. Often, this is relegated to tokenistic gestures rather than authentic partnerships. The aim of this national project is to use evidence-generated knowledge co-led by consumers, community members, researchers and clinicians to embed CCI in health and medical research and healthcare improvement.

**Methods:**

The Consolidated Framework for Implementation Research and the Learning Health System framework underpin the project to facilitate an iterative process to change systems and individual behaviour towards adoption of CCI in health and medical research and healthcare improvement. Key stakeholder groups include research translation centres, funding bodies, clinicians, professional staff involved in healthcare improvement, researchers and consumers and community members. To understand the attitudes, knowledge, beliefs, behaviours, system barriers and facilitators around CCI in health and medical research and healthcare improvement, semi-structured interviews and surveys will be conducted across key stakeholder groups. Template analysis and descriptive statistics will be used to report data from the national survey respectively prior to triangulation of data. Findings will be reported through traditional scientific outputs such as conference presentations and peer-reviewed publications. Other anticipated outputs include policy briefs, organizational implementation toolkits and resources and a co-designed digital knowledge hub to support individuals with implementation and scale up across stakeholders.

**Discussion:**

This study will build on considerable stakeholder engagement and prior priority-setting and includes broad and detailed consideration of perspectives from diverse stakeholders at a national level. Robust methodological frameworks, co-design and partnership with stakeholders will be used to inform resources to support systems change to facilitate CCI in health and medical research and healthcare improvement. Ethics approval was obtained from Monash Health (RES-23–0000-275Q).

## Background

Patients and other members of the public, referred to as consumers and community members in Australia, provide unique perspectives from their experiences to shape health and medical research and the healthcare system [1, 2]. Furthermore, as funders and beneficiaries of research and healthcare, consumers and community members deserve to be recognized and involved as key stakeholders in research and healthcare improvement projects from conception and delivery through to implementation to ensure that the outcomes meet their needs [1, 2]. Internationally, there has been a gradual increase in the uptake of patient and public involvement (PPI) in health and medical research and healthcare, which is now prioritized at policy, organizational and individual levels [3, 4].

Following global recognition of the importance of PPI, there have been increased efforts within Australia to embed what is known as consumer and community involvement (CCI) in health and medical research and healthcare improvement. For example, partnering with consumers is one of the eight areas of the National Safety and Quality Health Service Standards clinical organizations need to achieve to receive accreditation [5]. In 2016, the National Health and Medical Research Council (NHMRC) and Consumers Health Forum of Australia (CHF) developed a statement on CCI in health and medical research to guide research institutions, researchers and consumers and community members on CCI in health and medical research [2]. In 2023, another key Australian funding body, the Medical Research Future Fund (MRFF), released guiding principles for CCI in research to foster collaboration between consumers, researchers, research organizations and other health and medical research stakeholders [6]. Despite funding organizations providing CCI values and principles and increasing policy mandates to include CCI in grant applications, there is a lack of system processes and structures in place needed to support CCI [7, 8]. Consequently, CCI is often not an authentic, meaningful and active partnership [9].

CCI has become a priority area for the Australian Health Research Alliance (AHRA), who are a collective of NHMRC-accredited research translation centres, charged with embedding research into healthcare [10]. These evidence-based entities integrate and are sustainably funded by partners, including health services, research centres and universities [11]. Together, members across all AHRA Centres include over 90% of researchers and 86% of acute health care services in Australia [11]. Here, AHRA has partnered with the CHF and other key stakeholders in this work. Previous work by the AHRA includes two national surveys and co-design workshops with consumers and community members, researchers and clinicians which identified the need for broader CCI adoption and implementation, including a digital platform for stakeholders to share CCI resources and expertise and build capacity, referred to as a “knowledge hub” [12, 13]. A second national AHRA CCI survey undertaken in 2020 was designed to identify and prioritize resources and features to include in the digital knowledge hub [12]. The survey garnered 201 responses across Australia, including 41% from researchers, 31% from consumers, and 16% from clinicians and the remaining participants coming from management positions [12]. Six co-design workshops held across Australia were attended by 85 consumers, researchers and clinicians who explored and refined the information and priorities reported in the survey [12]. Finally, three stakeholder feedback workshops, attended by 45 consumers, researchers and clinicians, were conducted to refine the features and functions of the digital knowledge hub [12]. Furthermore, 34 interviews were completed with individuals from healthcare improvement and health and medical research settings to understand CCI capacity-building needs and the content for inclusion in the knowledge hub [13]. Four key areas of content identified from interviews included how to practically embed CCI in projects, how to connect and work with consumers and community members and how to progress CCI understanding and uptake [13].

The overarching purpose of the current research project is to apply co-design and implementation research methods to co-produce knowledge and resources with consumers, researchers and clinicians. Resources developed will detail the who, what and how to effectively embed CCI in healthcare improvement and health and medical research within Australia, including strategies at policy, organizational and individual levels. The specific aims of the project are to:

1. Apply implementation and behavioural science to understand how to change systems and individual-level CCI behaviours;
2. Identify and engage all relevant stakeholders;
3. Generate and synthesize evidence and evaluate existing CCI networks and digital resources;
4. Co-design, implement and evaluate CCI network models for engaging and upskilling consumers;
5. Co-design, implement and evaluate evidence-based policy and organizational toolkits and resources as well as an innovative digital knowledge hub with CCI training, tools and resources;
6. Evaluate the overarching project and generate broader organizational and policy implementation knowledge.

This work is funded by the Medical Research Future Fund through a competitive grant and through partnership across engaged stakeholders. Aims that are unable to be met within the 2-year grant timeline, including evaluation, will be embedded within the AHRA Research Translation Centres, which have been sustained over the past 10 years with ongoing partner and government funding.

## Methods and analysis

### Patient and public involvement

In addition to the preceding co-design work undertaken by AHRA [12, 13], CCI underpins this project. Founded on and prioritized through CCI and broad stakeholder involvement, the CCI advocate and consumer lead of the project (A.C.) has partnered with the research team from the conception phase. In addition to co-producing the research questions, A.C. provides key oversight and input into project governance and day-to-day project delivery. In her external advocacy roles and networks, including funders (NHRMC), A.C. will engage stakeholders and champions for project impact through delivery, progress and translation of findings to stakeholders.

The project steering committee consists of the consumer lead, and two other consumers, one of whom represents CHF, alongside the research team. Together, the consumers partner in providing governance and oversight across the project through a quarterly steering committee and fortnightly project meetings as well as email communication. Consumers from the steering committee will be involved with the research team co-designing interview guides and the national survey. A consumer advisory panel consists of 10 members from diverse backgrounds who have an interest in supporting CCI in health and medical research and healthcare improvement. This panel will inform and guide the project, by shaping outcome measures and evaluation processes, through workshops, interviews, co-design of a CCI network model and digital knowledge hub, by being consumer representatives in the project case studies. The consumer advisory panel and the AHRA Research Translation Centres will draw upon their extensive CCI networks throughout, including people from marginalized communities such as those living in regional and rural areas, Indigenous communities and culturally and linguistically diverse populations.

Aside from the AHRA Research Translation Centres, their partners and consumers and community members and representatives, other key stakeholders in this project include national funding bodies of health and medical research, health and medical researchers, clinicians and professional staff involved in healthcare improvement.

### Implementation and behavioural science frameworks (aim 1)

The Consolidated Framework for Implementation Research (CFIR) will be used to map stakeholders and contextualize CCI within the current Australian system and to guide implementation and adoption of CCI across healthcare and research [14–16]. Spanning across five core domains, CFIR comprises (i) the outer context (national research funding bodies such as the NHMRC, MRFF, consumer organizations such as CHF, policies, community, stakeholder and funder attitudes and expectations); (ii) the inner organizational context (research translation centres, research institutions and health services); (iii) the individuals in the system, who are the researchers, clinicians and consumers and community members and their respective roles in CCI; (iv) the evidence-based interventions to be implemented across the system; and (v) the process to implement this [14, 15]. At a systems level, we will explore implementation factors for CCI at the outer and inner organizational levels. This includes interventions, such as policies, awareness strategies/approaches, support tools, CCI networks and education opportunities, which can enhance genuine and impactful CCI at an individual level through changed knowledge, attitudes and behaviours. At the individual level, the Capability, Opportunity, Motivation and Behaviour (COM-B) model [17], which sits within the CFIR, will be applied to understand behavioural drivers of an individual’s actions, within the context of the inner and outer settings, to deliver best practice CCI [17].

While the CFIR framework can be used to map CCI stakeholders and their responsibilities in healthcare improvement and health and medical research, its implementation component oversimplifies the processes required for change in a complex system [14]. Specifically, within the implementation processes domain of CFIR, the construct for implementation is summarized by “doing”, which fails to capture the iterative dynamic process required to foster complex systems change [14, 15]. Therefore, here we apply the Learning Health System framework (LHS), as an evidence-based, co-designed model for iterative process to facilitate complex systems behaviour change for implementation [18–20]. The LHS captures, generates and leverages evidence across four broad quadrants including from stakeholder, research, practice/data and implementation to drive evidence into practice and towards systems and individual behaviour change, in this case in CCI [18–20].

Both the CFIR and the LHS incorporate the reach, efficacy, adoption, implementation and maintenance (RE-AIM) evaluation framework [21, 22]. The RE-AIM framework will be applied here to evaluate CCI networks and the digital knowledge hub. Finally, to co-design the digital knowledge hub, we will use the UK Design Council’s Double Diamond process. This co-design process will involve understanding users and their needs, define the area of focus, design and develop ideas and generate potential solutions to deliver the digital knowledge hub through robust usability testing [23].

**Fig. 1:**
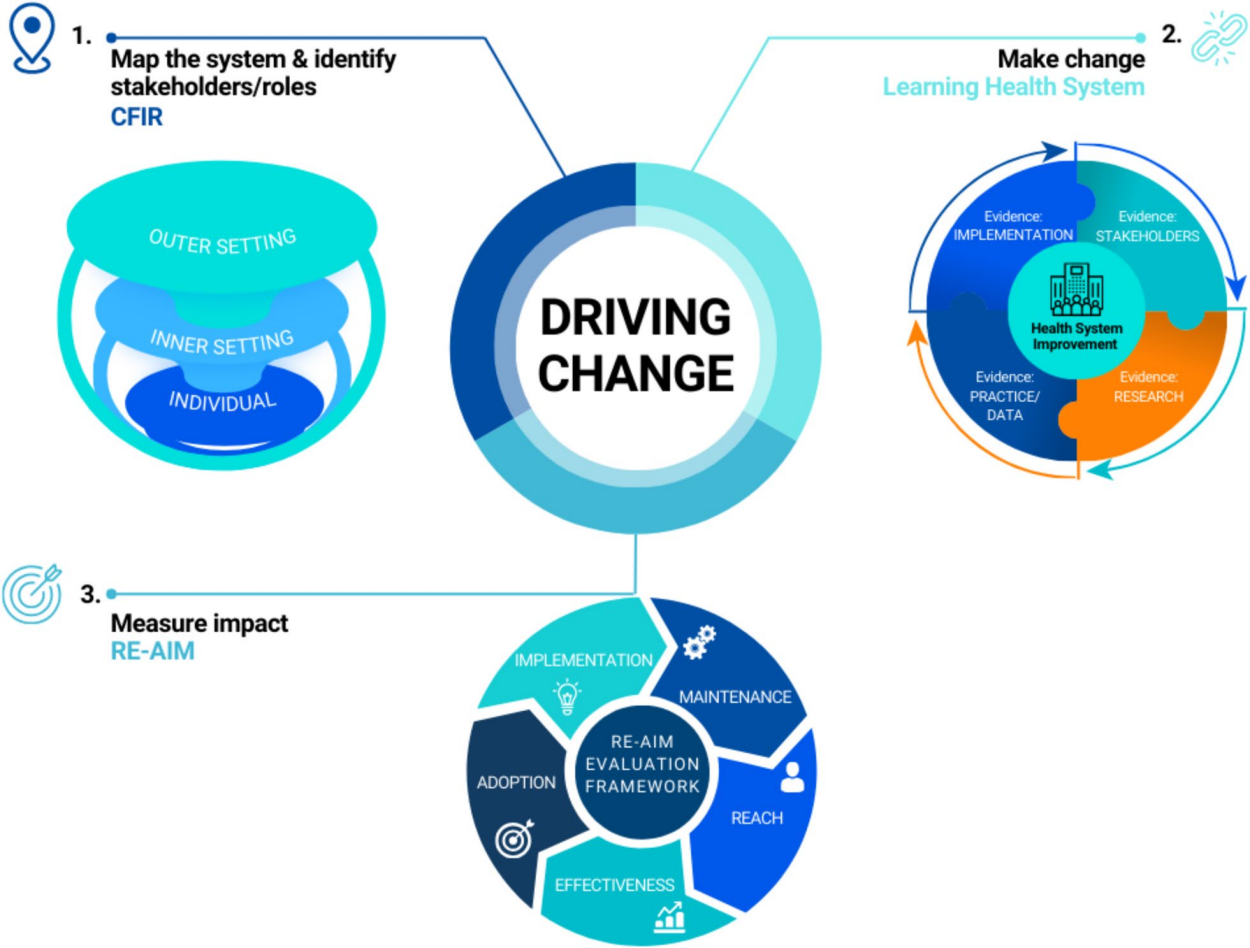
Interplay between the Consolidated Framework for Implementation Research^15^, Learning Health System ^19^ framework and reach, effectiveness, adoption, implementation and maintenance^22^ framework proposed to drive systems change in CCI

Figure 1 shows the overarching process of driving change towards CCI uptake in healthcare improvement and health and medical research using the CFIR, LHS and RE-AIM frameworks.

### Stakeholder engagement (aim 2)

**Fig. 2:**
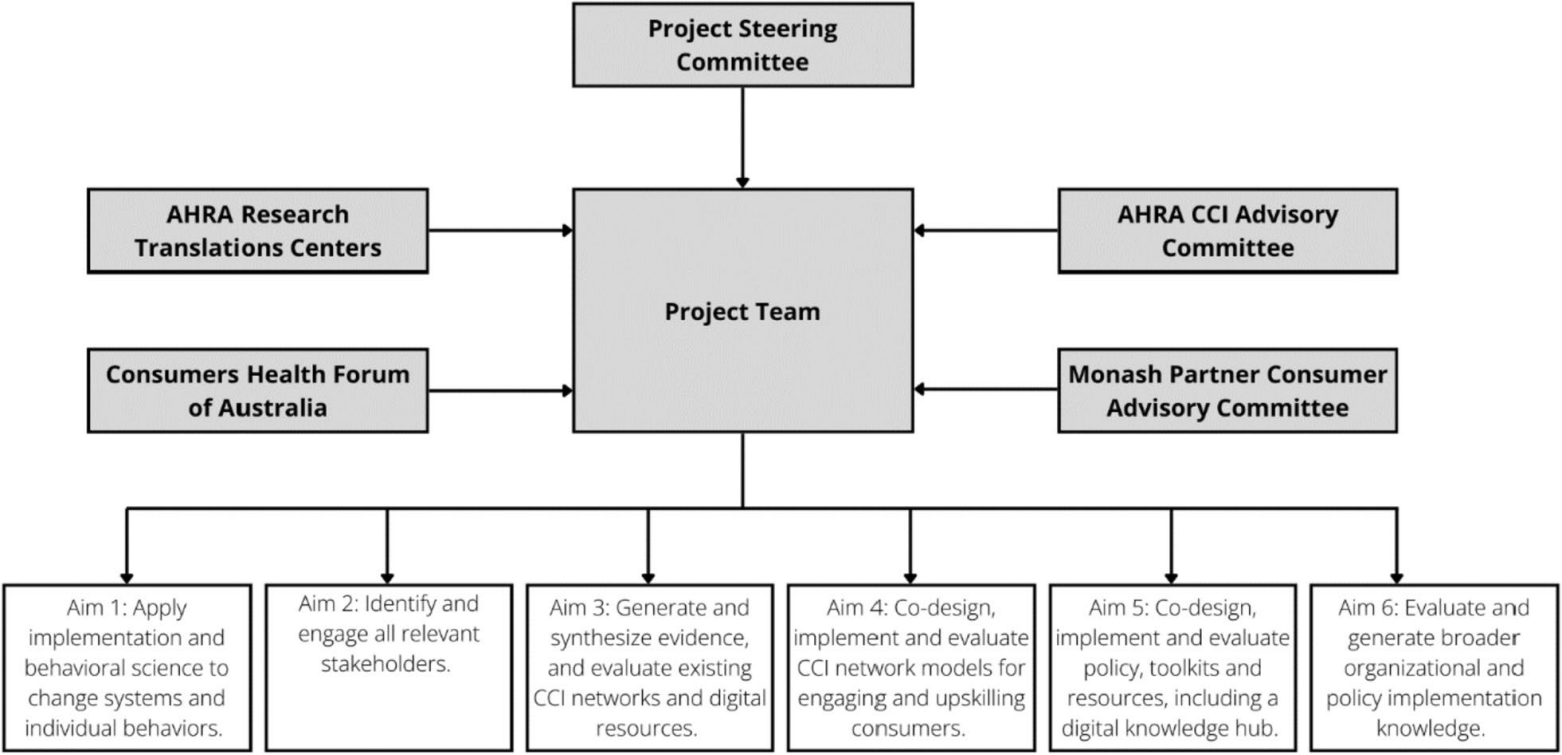
Overall project governance structure

A stakeholder matrix based on the five CFIR constructs will be used to comprehensively identify diverse key stakeholders [14]. Additionally, a broad range of engagement strategies will be used to reach key stakeholders, drawing upon networks known to the research team and outreach into community groups to ensure a broad and representative sample that is reflective of the health and medical research researchers, healthcare improvement staff and clinicians and a multicultural Australian population. Consumers are also invited to oversee the governance of the project by responding to an expression of interest through Monash Partners and CHF and a brief informal interview with shortlisted candidates. The overall project governance structure is highlighted in Fig. 2.

### Data collection tools & analysis

A range of collection tools will be used to collect data. A scoping review will be conducted and will synthesize evidence on building, growing and sustaining consumer networks, aligned to aim 3. Semi-structured interviews and a national survey will use co-designed interview guides and questions informed by the implementation science frameworks, stakeholder feedback and evidence synthesis. Recruitment and dissemination will target the AHRA Research Translation Centres, CCI Networks, CHF and other identified key stakeholders, with targeted efforts to reach diverse community groups. These will be conducted to gather data around current CCI organizational and systems factors and practices; knowledge, beliefs and attitudes towards CCI; and the structures and processes needed to build, grow and sustain CCI networks. All semi-structured interviews will be audio recorded and transcribed. Template analysis will be utilized to thematically analyse qualitative data [24]. Template analysis is a flexible process in which new information can be easily incorporated or adjustments made [25]. Development of the final template is an iterative process in which modifications are possible and expected [25]. Given that there is no limit on the number of iterations of the template, new concepts can be easily captured and incorporated as they arise. As part of this process, two researchers will develop a coding template by defining themes a priori after coding five transcripts from each stakeholder group each. The two researchers will then come together to jointly develop a coding template that will be finalized with consumer and project team input. The remaining dataset will then be coded to this template.

**Fig. 3:**
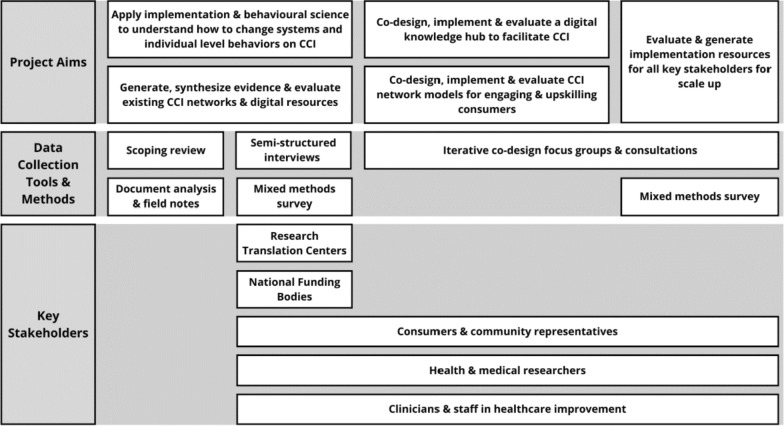
Data collection methods used throughout the project aligned to key stakeholder groups

Quantitative analysis will be descriptively analysed and summarized. This data will be supplemented by document analysis (terms of references, vision and mission statements, organizational structure and policy document, etc.) and field notes collected during stakeholder engagement. Evaluation using the RE-AIM framework will be applied to case studies including an existing mature CCI network, as defined by broad reach and extensive sustained membership. Here, data collection will include workshops, focus groups and semi-structured interviews. Data will contribute to understanding best practice structures and processes for effective and successful CCI networks. Data collection tools used throughout the project are summarized in Fig. 3.

### Methodology

**Fig. 4:**
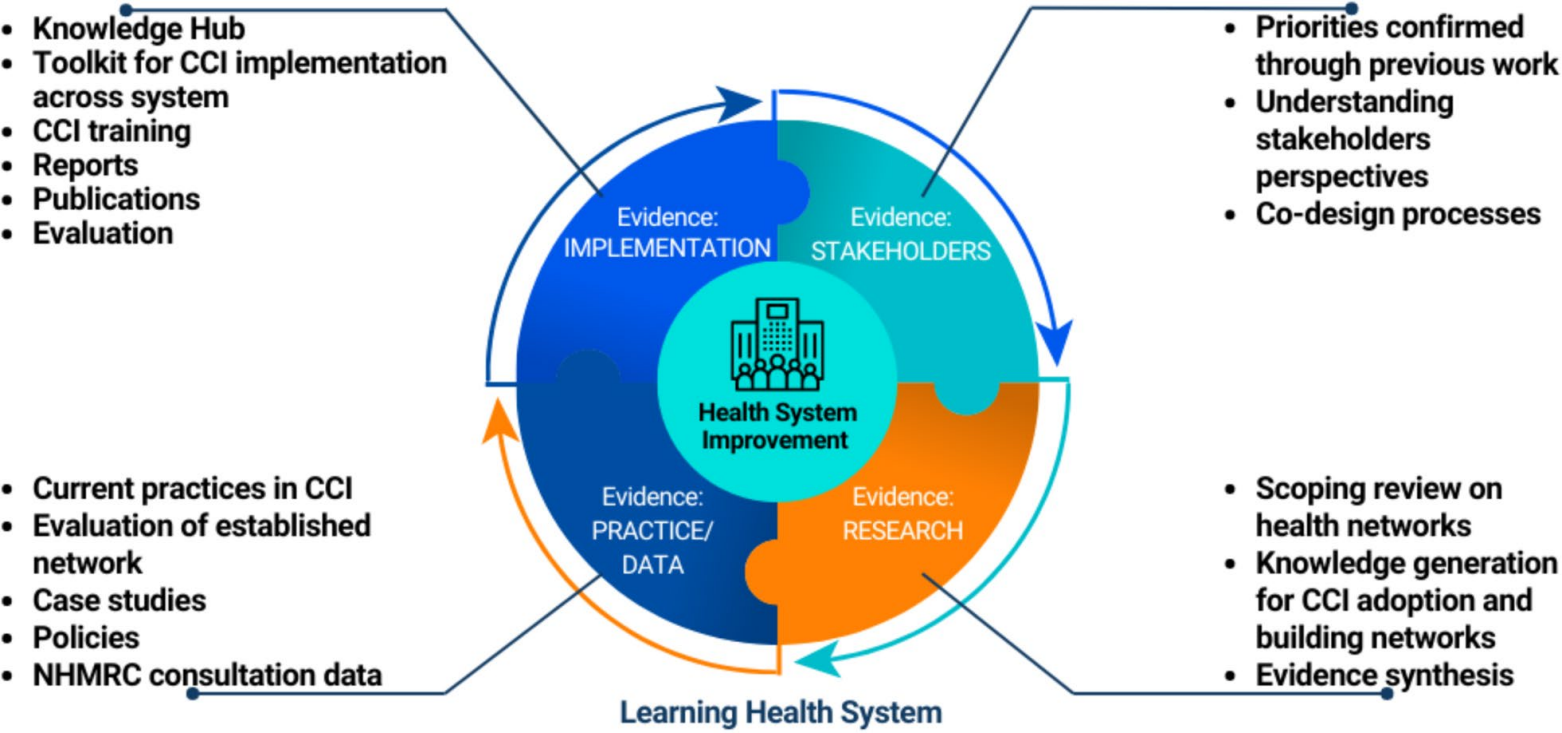
Methodology underpinning the project mapped against the Learning Health System^18^

The methodology of the project is aligned with the LHS framework as outlined in Fig. 4 and will be discussed in further detail according to the quadrants of (i) stakeholder engagement and priority-setting (aim 2), (ii) evidence synthesis and knowledge generation (aims 4–5), (iii) current practice and data (aims 4–5) and (iv) implementation (aims 4–6).

### Quadrant 1: Stakeholder engagement and priority-setting

The first quadrant from the LHS framework emphasizes priority-setting and understanding stakeholder’s perspectives to ensure any program developed is relevant to them and more likely to meet their needs [18]. Priorities for this project were informed by nation-wide surveys, focus groups and interviews undertaken by AHRA as described earlier [12–26]. Prioritization of CCI and strategies to increase its adoption across healthcare improvement and health and medical research, such as a digital knowledge hub, was informed by a previous national survey and a 1-day workshop [12]. Following this, a second national survey and co-design workshops were held to scope features and functions to include in the digital knowledge hub [13]. Additional interviews were held to understand the educational needs from people embedded in healthcare improvement and health and medical research, which informs the content within the digital knowledge hub [13]. Within the current project, stakeholders will be engaged to reconfirm priorities, further refine the design of and content for the digital knowledge hub and co-design evaluation outcomes for the CCI network and knowledge hub.

Key stakeholders for the current project have been identified using the CFIR model to maximize engagement and impact across systems levels [14]. Decision-makers from organizations leading research policy, conduct and grant funding across Australia will provide an outer setting perspective on CCI in the health and medical research context. CCI leads from AHRA Research Translation Centres will provide insights across both outer and inner settings of the system in which CCI operates. These CCI leads will also provide insights as individuals acting within the outer and inner settings on the adoption, attitudes, beliefs and behaviours around CCI. Consumer and community members, clinicians, professional staff involved in healthcare improvement and health and medical researchers will be purposively sampled to achieve diversity in backgrounds, context and experiences from across Australia. Data on their insights into, and experiences with, CCI in healthcare improvement and health and medical research will be collected. A consumer advisory panel will be established to ensure consumer involvement at each stage of the project. The panel will be chaired by a consumer and engaged in the project through regular meetings, email discussions and other ad hoc opportunities. In conjunction, a steering committee will provide oversight to the project, which includes the consumer-lead research investigator.

### Quadrant 2: Evidence synthesis and knowledge generation

The second quadrant of the LHS framework focuses on generating knowledge and synthesizing findings with existing research [18]. To understand structures and processes required to build, grow, sustain and evaluate CCI networks, a scoping review and evaluation of an existing mature CCI network will be conducted using data collection methods described in Fig. 3. Findings from the scoping review, evaluation of existing CCI networks, document analysis and field notes will be mapped to the CFIR framework to understand the influences, connections and interactions between and within the various settings and individuals.

To improve understanding about individual knowledge, attitudes and beliefs to influence systems and behaviour change in CCI adoption, a national survey and semi-structured interviews will be conducted as outlined in Fig. 3. Interviews and surveys will be informed by the CFIR [14–16] and COM-B frameworks [17]. The COM-B model, which is also nestled within the CFIR framework, guides understanding of behaviour and drivers of an individual’s ability to put knowledge into action within their environmental support to develop behaviour change interventions [17]. Additionally, the CFIR framework supports exploration of barriers and facilitators from a broader perspective such as the outer setting (for example, policy mandates) and inner setting (for example, research institution environments) and at an individual level to support CCI adoption.

Collectively, data from the scoping review, CCI network evaluation, consumer advisory panel feedback and field notes will be synthesized to develop best practice guidelines for CCI network models and resources for broader implementation. Findings from the semi-structured interviews and surveys will inform key messaging and resources within the knowledge hub for CCI adoption across stakeholder groups. Once these findings have been embedded into the early design phase of the knowledge hub development, an iterative series of co-design workshops will be held to refine the content and design of the platform.

The knowledge hub content creation, prototype co-design, navigation, user acceptability and usability testing and refinement will follow best practice digital tool development processes, as per our prior work. Using the UK Design Council Double Diamond co-design process [23], co-design workshops and further individual feedback consultations with diverse stakeholder groups will be held to develop the digital knowledge hub. The Double Diamond model fosters co-design through four phases: (i) discover – working with stakeholders to understand the problem; (ii) define – focusing on specific problem areas; (iii) develop – identifying solutions with stakeholders such as consumers, researchers, clinicians and digital design experts; and (iv) deliver – testing and evaluating solutions [23]. After each co-design workshop, discussions and field notes will be synthesized into action items to build the next iteration. Once the design infrastructure has been finalized, a clickable prototype will be developed, and individual consultations will be held with purposively sampled participants from stakeholder groups, including people involved in previous co-design workshops and individuals seeing the prototype for the first time. Feedback will be incorporated into the final version of the knowledge hub before it becomes publicly available.

### Quadrant 3: Current practice and data

In the third quadrant of the LHS framework, understanding and integrating current practice and data are crucial to support iterative change processes [18]. In line with this, evaluation of an established CCI network, based in Western Australia, will capture historical and current practice through focus groups, interviews, document analysis and field notes to inform the co-design of best practice guidelines and resources for implementation of other CCI networks.

Research case studies across four different clinical domains from New South Wales and Victoria will also capture data from real-world practice and projects. Research case studies will be informed and supported by the digital knowledge hub, backed by a CCI network where relevant, as well as CCI messaging refined from the survey and interviews findings.

The implementation science and CFIR approaches address systems, organizational and individual perspectives and hence explore and generate evidence on systems change across state and federal activities. Knowledge hub content and implementation resources will reflect findings and recommendations from the survey and semi-structured interviews that capture current CCI practice in healthcare improvement and health and medical research. Findings from a national CCI consultation conducted to revise the NHMRC and CHF Joint Statement on CCI in Health and Medical Research will also be drawn upon to refine the knowledge hub content and its implementation resources.

### Quadrant 4: Implementation

As part of implementation in the fourth quadrant of the LHS, evaluation of co-design processes, the knowledge hub and overall project will be undertaken using the RE-AIM framework [21, 22]. Evaluation outcomes for the knowledge hub will include the number and reach of active users, the impact from using the digital knowledge hub, the process and structure to achieve uptake from members and the user experience of the knowledge hub. Evaluation outcomes for the consumer advisory panel will be co-designed with members to ensure relevancy and ongoing sustainability.

**Fig. 5:**
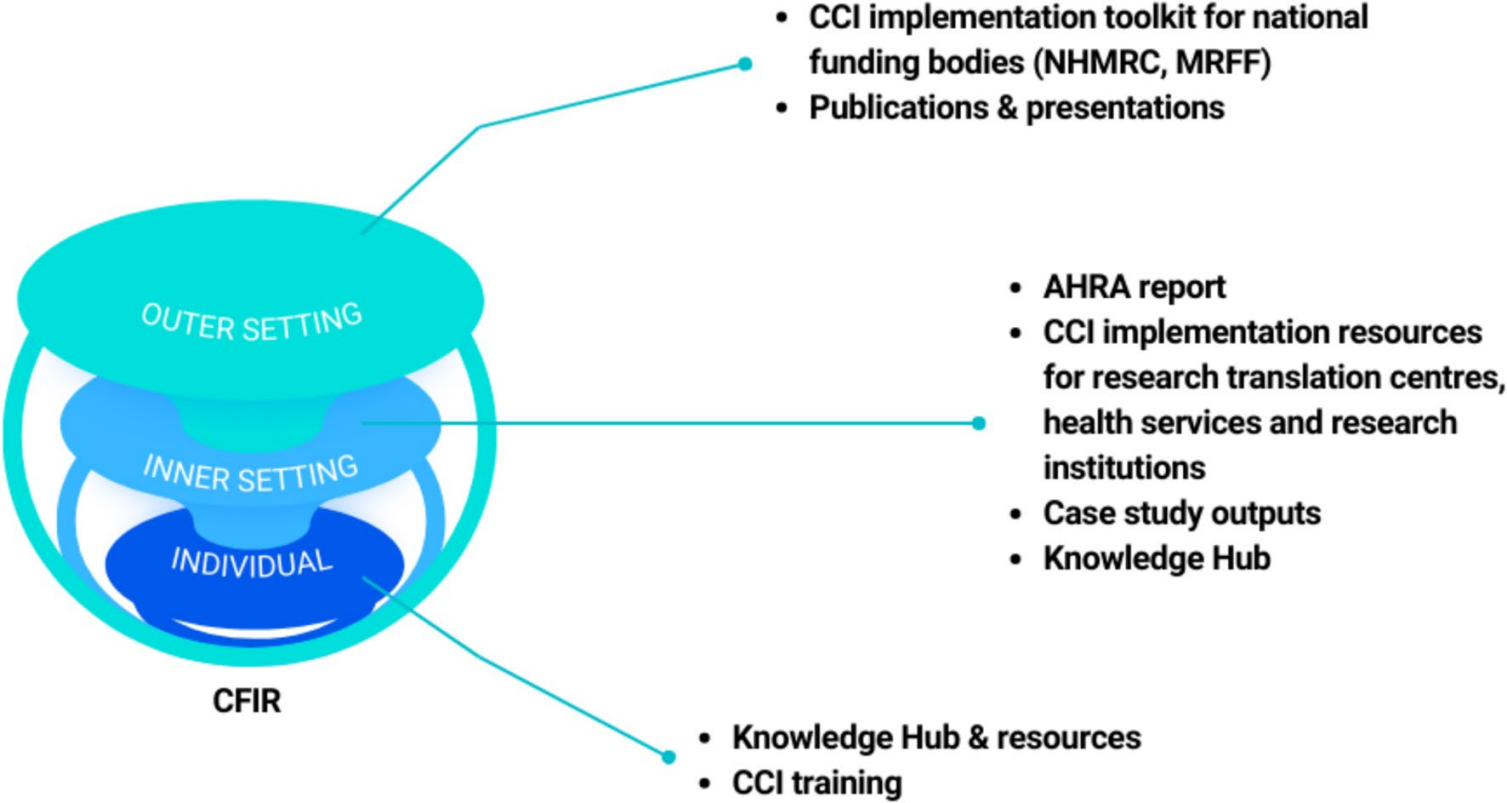
Implementation resources outputs mapped against the Consolidated Framework for Implementation Research^15^

Implementation resources will be co-developed and mapped to the CFIR framework to enable a targeted approach across each level of the system that CCI operates in for health and medical research and healthcare improvement [14, 15]. Understanding the role that each key stakeholder holds will provide an in-depth understanding of the support and changes to the CCI system that need to be undertaken to promote its adoption [14, 15]. The generation of evidence for implementation will inform the digital knowledge hub and the development of a CCI toolkit and translation strategies for organizations to successfully adopt CCI into daily practice as highlighted in Fig. 5.

Ultimately, evaluation of the knowledge hub and the key strategies developed to support CCI adoption will identify areas for development, thereby continuing the cycle of the LHS. Implementation and evaluation will be co-designed, including outcomes, and is anticipated to leverage the AHRA Research Translation Centres, CHF, funding bodies and project stakeholder involvement. The RE-AIM framework will be applied across reach (tool use and uptake), efficacy (outcomes to be co-designed but likely to include review of funding agency proposals for trends in CCI quality over time, consumer and researcher surveys and interviews), adoption (integration into organizational processes), implementation (fidelity of evidence-based processes and tools) and maintenance of study outputs over time. An evaluation of the overall co-design project will take place informed by the implementation science and RE-AIM frameworks. Mixed methods data collection will generate new knowledge on co-design, stakeholder and consumer engagement and implementation of effective resources and strategies at scale.

## Ethics and dissemination

Ethics approval was obtained from Monash Health Research Ethics Committee (RES-23-0000-275Q). Findings will be co-presented at conferences and co-authored with consumers in peer-reviewed publications. Alongside traditional scientific outputs, co-produced summary reports will be provided to national research funding bodies and research translation centres to inform policy and CCI network development and support implementation of the digital knowledge hub. Resources such as infographics will be co-designed with stakeholders and be disseminated to the public, alongside peer-reviewed publications and summary reports, via the digital knowledge hub.

## Discussion

Healthcare improvement and health and medical research interacts within a highly complex and dynamic system. Implementing complex systems change can present major challenges. Here, we will generate new knowledge and co-design, implement and evaluate evidence-based resources to improve CCI across Australia. CCI is embedded within and throughout the project and key stakeholders involved. Robust implementation science frameworks, co-design approaches and data collection are incorporated. At a systems level, considerations include the outer (policy and funders) and inner organizational levels. Interventions, such as policies, awareness strategies/approaches, support tools, CCI networks and education opportunities can enhance genuine and impactful CCI at an individual level through changed knowledge, attitudes and behaviours.

Increasing recognition of the value of CCI in healthcare improvement and health and medical research has led to the need to prioritize CCI implementation. Without adequate support and guidance, genuine and meaningful CCI is unlikely to occur. Optimizing implementation requires evidence on the who, what and how, as highlighted through the LHS quadrants. Here, we present the protocol for a large-scale, systems-level national initiative to deliver on policy and stakeholder priorities for integrated genuine CCI across the “who” (stakeholders across the outer, inner and individual levels), the “what” (networks, the digital knowledge hub, resources, and tools) and the “how” (policies, processes and strategies at all levels). This work extends beyond CCI implementation as an exemplar of large-scale systems change with broader learning relevant to healthcare improvement and health and medical research.

## Data Availability

No datasets were generated or analysed during the current study.

## Abbreviations

PPI: Patient and Public Involvement
CCI: Consumer and Community Involvement
NHMRC: National Health and Medical Research Council
CHF: Consumers Health Forum of Australia
MRFF: Medical Research Future Fund
AHRA: Australian Health Research Alliance
CFIR: Consolidated Framework for Implementation Research
COM-B: Capability, Opportunity, Motivation and Behaviour model
LHS: Learning Health System
RE-AIM: Reach, Efficacy, Adoption, Implementation and Maintenance framework
